# Mediating effects of rumination on insomnia in cancer survivors: Influences of cancer‐related fatigue, fear of recurrence, and psychological distress

**DOI:** 10.1002/cam4.70189

**Published:** 2024-09-21

**Authors:** Omid Amani, Mohammad Ali Mazaheri, Mona Malekzadeh Moghani, Fariba Zarani, Rasool Hamidi Choolabi

**Affiliations:** ^1^ Department of Psychology Shahid Beheshti University Tehran Iran; ^2^ Department of Radiation Oncology Shahid Beheshti University of Medical Sciences Tehran Iran; ^3^ Department of Psychology Ahrar Institute of Technology and Higher Education Rasht Iran

**Keywords:** cancer, cancer‐related fatigue, fear of cancer recurrence, insomnia, psychological distress, rumination

## Abstract

**Background:**

While advancements in cancer treatments have improved survival rates, they also lead to adverse effects such as insomnia, which significantly impacts survivors' sleep quality.

**Objective:**

This study explores the influence of cancer‐related fatigue (CRF), Fear of Cancer Recurrence (FCR), and psychological distress, with rumination serving as a mediating factor, on the insomnia experienced by cancer survivors.

**Methods:**

The study involved 220 cancer survivors attending Shohada‐e‐Tajrish Hospital's oncology center in Tehran, Iran. Participants were selected through convenience sampling and completed several questionnaires: the Insomnia Severity Index, Fear of Cancer Recurrence Inventory, Cancer Fatigue Scale, Kessler Psychological Distress Scale, and Rumination Response Scale.

**Results:**

The results showed that the tested model had a good fit, and the correlation matrix demonstrated significant positive correlations between CRF (0.46), FCR (0.15), psychological distress (0.55), and rumination (0.42) with insomnia in cancer survivors (*p* < 0.05). Notably, CRF (B = 0.356, *p* < 0.001) and psychological distress (B = 0.339, *p* < 0.001) affect insomnia both directly and indirectly through mediation by rumination, while the impact of FCR on insomnia was indirectly significant (B = 0.73, *p* < 0.05).

**Conclusion:**

The findings suggest that interventions focused on managing rumination could be potential targets to alleviate insomnia and improve the sleep quality of cancer survivors.

## Graphical abstract

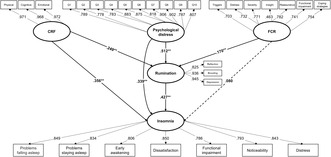

## 1 INTRODUCTION

Cancer is a chronic and non‐contagious disease encompassing a group of diseases characterized by uncontrolled and progressive cell multiplication and abnormal tissue development.^1^ It is known as the second‐leading cause of death worldwide^2^ and the third‐leading cause of death in Iran.^3^ Although the most widely adopted treatments, such as surgery, hormone therapy, radiation therapy, and chemotherapy, have improved patients' longevity, these methods of treatment also leave behind several damages that, by diminishing quality of life, jeopardize any benefits of more prolonged survival.^4^ Insomnia is a common psychological consequence frequently observed among cancer survivors following their treatment.^5^ Research indicates that while two‐thirds of cancer survivors suffer from a range of sleep disorders such as restless leg syndrome and sleep apnea,^6^ the prevalence of insomnia, which is regarded as a psychobiological disorder, is three times higher in this group compared to the general population.^7^

Insomnia is a sleep disorder that manifests through a range of symptoms, including difficulty falling asleep, frequent awakening throughout the night, difficulty maintaining sleep, early morning awakening with an inability to fall back asleep, and daytime sleepiness. These symptoms occur at least three nights per week for a minimum of 3 months.^8^ Studies have shown various reasons for insomnia in cancer survivors. Apart from the biological symptoms associated with cancer, such as hot flashes, night sweats, and sudden onset of menopause due to chemotherapy and hormone therapy,^9^ non‐biological factors play a critical role in the development and persistence of insomnia. These factors are diverse and significantly contribute to the perpetuation of insomnia. The psychological distress associated with the diagnosis and intensive curative treatments renders patients at risk for long‐term effects, including insomnia, which can persist well beyond the active treatment phase.^10^

The origins, contributing factors, and underlying mechanisms of insomnia are complex and not fully understood, particularly among cancer survivors. This complexity is framed by the Three‐Factor Model (3P), which categorizes the elements of insomnia into predisposing, precipitating, and perpetuating factors specific to this population.^10^, ^11^ Predisposing factors, such as a tendency toward rumination and worry, increase vulnerability to insomnia. Precipitating factors include the significant emotional and physiological stresses of diagnosis and treatment. Perpetuating factors, such as irregular sleep schedules and poor sleep hygiene, maintain the chronic nature of the disorder.^10^

One of the psychological factors that has been mentioned in relation to the persistence of insomnia in cancer survivors is cancer‐related fatigue (CRF).^12^ CRF describes the prevalent experience of fatigue among cancer survivors.^13^ Research suggests that between 72% and 99% of cancer survivors experience some form of fatigue related to the disease.^14^ The National Comprehensive Cancer Network defines CRF as “a distressing, persistent, subjective sense of physical, emotional, and/or cognitive tiredness or exhaustion related to cancer or cancer treatment that is not proportional to recent activity and interferes with usual functioning.”^15^ Studies indicate that the experience of CRF is different from the experience of fatigue in non‐cancer patients,^16^ and it is more severe, disabling, and persistent than normal fatigue and does not improve with rest.^17^

Another common and influential factor in the sleep quality of cancer survivors is the worry about the side effects of the disease, the prognosis, and the fear of recurrence (FCR),^18^ which can be distressing for the individual from various aspects and lead to psychological distress.^19^ FCR is defined as fear and concern about the recurrence or progression of cancer in the involved organ or other areas of the body of the cancer survivor.^20^ A prominent feature of this fear is the persistent and high levels of preoccupation, worry or excessive sensitivity to physical symptoms that last for at least 3 months and cause psychological distress in the individuals by interfering with their daily functioning.^21^ Psychological distress indicates a range of emotional elements, including symptoms of anxiety and depression.^22^ Although the intensity of psychological distress decreases over time, it remains chronic for a significant population of cancer survivors. Therefore, it affects different dimensions, such as treatment outcomes and post‐treatment quality of life.^23^

Among other cognitive variables affecting the psychological structure and insomnia of cancer survivors, which have gained special attention in recent decades, is rumination.^24^ Rumination is a series of persistent and recurring thoughts that revolve around a common theme, involuntarily entering consciousness and diverting an individual's attention from their intended subjects and goals.^25^ These passive repetitive thoughts impede problem‐solving and leads to an increase in negative affect.^26^ Studies suggest that rumination affects the cognitive structure of individuals, leading to increased negative emotions and feelings. This acts as a risk factor for a predisposition toward negative information and depression,^27^ which in turn leads to insomnia^24^ and reduced sleep quality.^28^ Several studies have examined the role of rumination in the development of symptoms like CRF, psychological distress, and insomnia among cancer survivors, including studies by Haque et al.^5^ Otte et al.^29^ Hwang et al.^30^ Thorsteinsson et al.^31^ and Slavish et al.^32^ Moreover, Reynolds‐Cowie and Fleming noted the lived experience of ruminative worry in cancer survivors, highlighting its significant impact on persistent insomnia.^33^

Harvey's cognitive model^34^ posits that ruminative thoughts followed by resultant worry activate the autonomic nervous system, leading to psychological distress and manifesting themselves in insomnia. Priede et al. also concluded in their study that rumination has a significant relationship with the psychological distress of cancer survivors.^35^ Furthermore, extensive research into the 3P Model of insomnia has significantly advanced our understanding of how psychological elements, particularly rumination, contribute to the persistence of insomnia among cancer survivors. This investigation acknowledges rumination's dual role in both predisposing individuals to and perpetuating insomnia.^10^ Notably, a study suggests that enhancing digital Cognitive Behavioral Therapy for Insomnia (dCBT‐I) to target rumination better could substantially improve treatment outcomes.^36^ Despite these insights, the specific roles of FCR, CRF, and psychological distress in perpetuating insomnia require further elucidation. Our study aims to deepen the understanding of how CRF, FCR, and psychological distress contribute to insomnia, with a particular focus on rumination's mediating role. By examining these interactions, we intend to identify targeted interventions that address the root causes of insomnia in cancer survivors, moving beyond mere symptom management. Considering what has been mentioned, we hypothesize the following:

1. CRF and FCR are positively associated with psychological distress and insomnia among cancer survivors.
2. Rumination mediates the relationship between CRF/FCR/psychological distress and the severity of insomnia in cancer survivors.

## 2 METHOD

The design of our study was descriptive and correlational, utilizing structural equation modeling to analyze the data. The participant pool consisted of cancer survivors referred to the oncology center at Shohada‐e‐Tajrish Hospital in Tehran, Iran, from May 2022 to January 2023. In alignment with Kline's guidelines a sample size of 3–5 respondents per questionnaire item is deemed sufficient, yet a minimum of 200 samples is advisable for robust analysis.^37^ To accommodate potential dropouts and enhance the validity of our findings, 220 participants were selected using a convenience sampling method.

### 2.1 Inclusion and exclusion criteria

Participants were required to meet the following inclusion criteria: a diagnosis of a single type of cancer, completion of at least 3 months post‐major treatment (surgery, chemotherapy, or radiotherapy), an age range of 20–60 years, ability to read and write, and provision of informed consent. The age range of 20–60 years was chosen to focus on the adult working‐age population, which is likely to have different psychosocial dynamics compared to those older than 60. Excluding adults older than 60 helps control for age‐related comorbidities and cognitive decline that could confound the study results. Our study involved participants who were considered cancer‐free, having completed primary cancer treatment modalities such as surgery, chemotherapy, and radiation. Additionally, participants undergoing maintenance therapies such as hormonal therapy or immunotherapy were included, as these are generally preventative and do not indicate the presence of active disease.

Exclusion criteria encompassed individuals with a history of multiple cancer types, diagnosed major depressive disorder, generalized anxiety disorder, bipolar disorder, schizophrenia, and other severe mental health conditions that could independently affect sleep patterns and psychological distress. Additionally, participants with chronic physical illnesses such as cardiovascular diseases, uncontrolled diabetes, chronic obstructive pulmonary disease, and other significant chronic illnesses were excluded to ensure these conditions did not confound the study results related to CRF and insomnia.

### 2.2 Data collection procedure

Following the approval of the research proposal and acquisition of necessary permissions, researchers visited the hospital to invite post‐treatment follow‐up patients to participate in the study. After obtaining informed consent, participants were invited to a controlled environment devoid of any potential disturbances that might affect the completion of the questionnaires. Initial participant information was collected, maintaining strict adherence to ethical research standards, including confidentiality. The questionnaires were administered in one session with the assistance of researchers, who also provided clarifications on any queries raised by participants. For any missing or incomplete data, multiple imputation methods were employed to maintain a total sample size of 220 participants for analysis.

### 2.3 Measures

#### 2.3.1 Insomnia Severity Index

This scale, developed by Morin et al. is designed to evaluate sleep problems. The respondent rates the severity of difficulties in^1^ falling asleep,^2^ sleep maintenance,^3^ nocturnal, and early awakenings,^4^ satisfaction with the current sleep pattern,^5^ sleep interference with daily functioning,^6^ noticeability of impairment due to the sleep difficulty, and^7^ distress caused by sleep problems. It consists of 7 questions. The scoring method for this tool involves rating the first 3 questions on a scale from 0 (never) to 4 (very severe) and the subsequent four questions from 0 (very satisfied) to 4 (very dissatisfied). The total score is calculated by summing up the scores of all items. Bastien et al. determined the construct validity of this instrument based on accuracy, severity, and satisfaction at 72%, and its reliability using internal consistency methods was found to be 74% and 87%.^38^ In Iran, Dastani et al. measured the reliability of the Persian version of the scale using Cronbach's alpha and reported a value of 0.72.^39^ The Cronbach's alpha coefficient is 0.92 for this study.

#### 2.3.2 Fear of cancer recurrence inventory

This self‐report instrument, developed by Simard and Savard to assess the FCR over the past month, consists of 42 questions scored on a Likert scale from 0 to 4. Only one item, “I believe that I am cured and that the cancer will not come back,” is reverse‐scored. In this questionnaire, significant clinical information related to the nature of the fear of cancer recurrence is obtained across seven components: triggers, psychological distress, severity, insight, reassurance seeking, functional impairment, and coping strategies. Scores for each component are summed to arrive at a total score, with higher aggregate scores indicating greater fear of cancer recurrence. In the original version of the questionnaire, an internal consistency of 0.75, test‐retest reliability of 0.58, and a Cronbach's alpha of 0.95 were reported, indicating high indices.^40^ In Iran, Kiarasi et al. conducted a study on the psychometric properties of the Persian version of this instrument and found its reliability, as measured by Cronbach's alpha, to be 0.93.^41^ The Cronbach's alpha coefficient for the present study is 0.84.

#### 2.3.3 Cancer Fatigue Scale

This scale, developed by Okuyama et al. is designed to measure CRF and consists of 15 questions, evaluating three subscales: physical (questions 1, 2, 3, 6, 9, 12, 15), cognitive (questions 4, 7, 10, 13), and emotional (questions 5, 8, 11, 14).^42^ The scoring method for this scale is based on a five‐point Likert scale ranging from 0 (not at all) to 4 (very much) for each question, with the total score indicating the patient's level of CRF.^43^ In this study, we used the Persian version of Cancer Fatigue Scale (CFS). The reliability of the Persian version of this instrument was calculated in a study by Aghayousefi et al. using the internal consistency method via Cronbach's alpha. The coefficients obtained were 0.88 for the physical component, 0.92 for the emotional component, 0.85 for the cognitive component, and 0.90 for the entire questionnaire.^44^ The Cronbach's alpha coefficient is 0.96 for this study.

#### 2.3.4 The Kessler Psychological Distress Scale

This instrument, developed by Kessler and Andrews in 2002, was created to identify mental disorders in the general population and is available in two versions: one with 10 questions and another with 6 questions. The 10 question version of this questionnaire is more effective in identifying mood and anxiety disorders, and in the current study, its Persian version has been used. The questions of this tool are rated on a four‐point Likert scale ranging from “never” to “always,” scored between 0 and 4. Therefore, the minimum score is 0, and the maximum is 40.^45^ To assess the validity of this instrument, Veldhuizen et al. compared it with the Composite International Diagnostic Interview for psychological disorders.^46^ They found that the tool's sensitivity to psychological problems was appropriate. It was revealed that Kessler's Psychological Distress Questionnaire has a high sensitivity for screening individuals with anxiety and depression. The reliability coefficient of Kessler's Psychological Distress Questionnaire was determined by Kessler et al. using both Cronbach's alpha and split‐half methods, resulting in values of 0.74 and 0.70, respectively, indicating good reliability of the questionnaire.^45^ The reliability of the Persian version of this instrument within the Iranian population was reported at 0.90 using Cronbach's alpha and 0.86 using the split‐half method.^47^ In the present study, the Cronbach's alpha coefficient for psychological distress is 0.95.

#### 2.3.5 Rumination Response Scale

This scale, developed by Nolen‐Hoeksema and Morrow to assess the extent of rumination, consists of 22 questions. This questionnaire includes three components: Reflection (questions 7, 11, 12, 20, and 21), Brooding (questions 5, 10, 13, 15, and 16), and Depression‐Related (questions 1 to 4, 6, 8, 9, 14, 17, 18, 19, and 22). Each question is scored on a four‐point scale from 1 (almost never) to 4 (almost always), with the total score ranging between 22 and 88. The psychometric assessment of this tool indicates that its reliability coefficient, as reported in retests, is 0.80, and the reliability determined through Cronbach's alpha method ranges between 0.88 and 0.92.^48^ The Cronbach's alpha coefficient is 0.95 for present study.

### 2.4 Statistical analysis

Statistical analyses were performed to ensure the rigor and validity of the questionnaire data. Confirmatory factor analysis was conducted to evaluate the validity of the questions, with factor loadings for all model indicators exceeding the minimum criterion of 0.40, thus establishing questionnaire validity. Convergent validity was confirmed through average variance extracted values, which exceeded the threshold of 0.50 for all variables. Reliability of the instruments was assessed using composite reliability and Cronbach's alpha, both of which indicated satisfactory reliability with values above 0.70. Discriminant validity was established using the Fornell‐Larcker criterion, and the absence of severe multicollinearity was confirmed by variance inflation factor scores, which were all below five.

**FIGURE 1:**
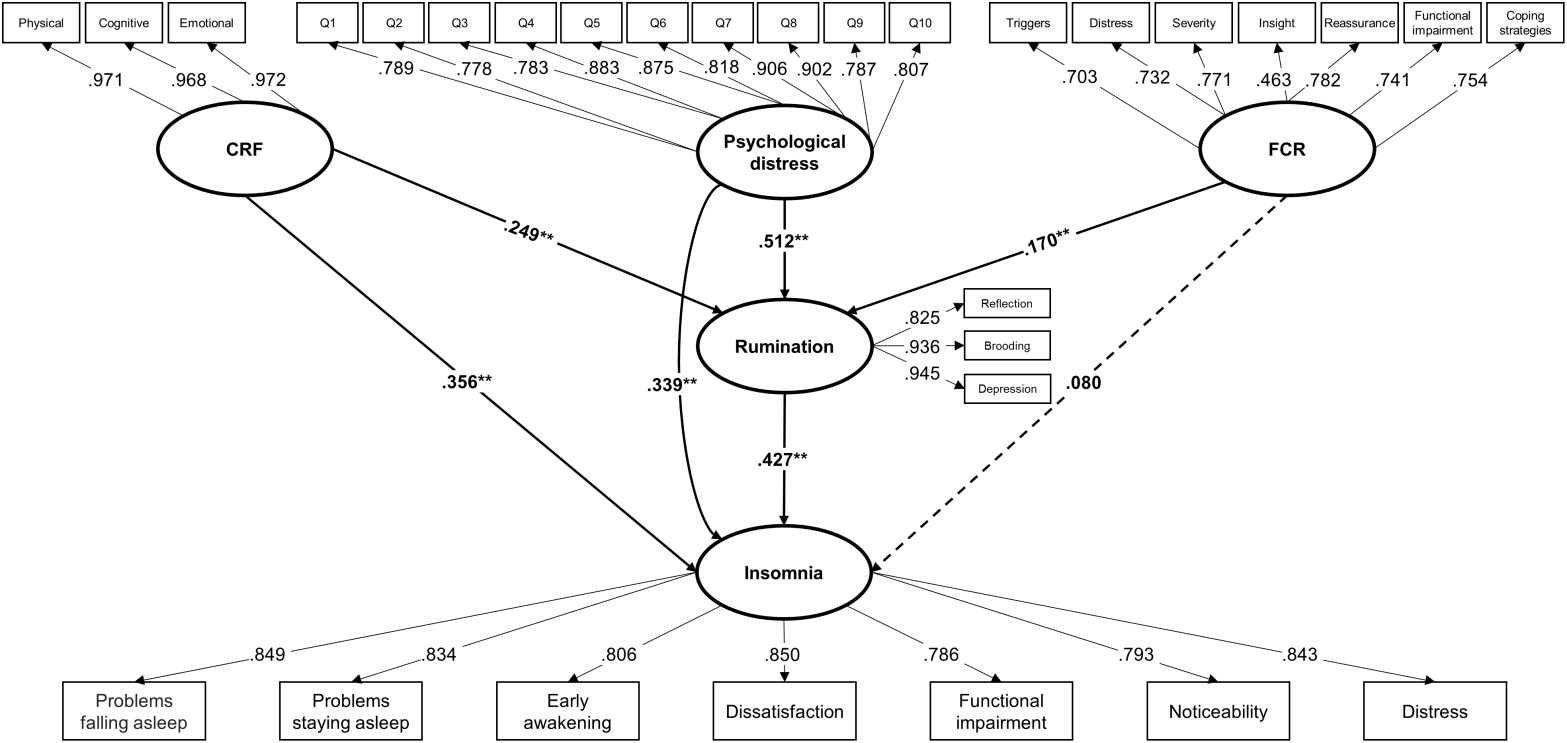
Experimental model in the case of standard path coefficients. CRF, cancer‐related fatigue; FCR, fear of cancer recurrence; Significance is marked with ** for *p* < 0.01.

Structural equation modeling (SEM) was analyzed using the Partial Least Squares (PLS) approach with SPSS v26 and Smart PLS3 software. The PLS approach was chosen due to its suitability for exploratory research and complex models with multiple constructs. The SEM included both measurement and structural components. The measurement model assessed the relationships between observed variables (indicators) and their underlying latent constructs, while the structural model evaluated the hypothesized relationships among the latent constructs. The model was tested using aggregate scores for each construct derived from the respective questionnaire items. Figure 1 illustrates the basic structure of the model, showing the pathways and relationships among the constructs. For model identification, the factor loadings for the first indicator of each latent construct (CRF, FCR, psychological distress, rumination, and insomnia) were fixed to 1.0, while all other factor loadings, path coefficients, and structural paths were estimated by the SEM algorithm, including paths between CRF, FCR, psychological distress, rumination, and insomnia.

## 3 RESULTS

The study sampled 220 cancer survivors, with 88.5% female and 11.5% male participants. Among these participants, 72.5% were married, 12.5% were widowed, 7.5% were divorced, and 6.5% were single. The mean age of respondents was 52.27 years (SD = 12.21). Educationally, 31.5% had less than a high school diploma, 33.7% had completed high school, 28.6% had an associate's or bachelor's degree, and 6.2% had obtained a master's or doctoral degree. Additionally, 74% of the cancer survivors had a history of surgery, while 26% had not undergone surgery related to their cancer. The average time elapsed since diagnosis was 49.91 months (SD = 44.92), with the average time since the last treatment being 26.52 months (SD = 28.71). Table 1 provides a detailed description of the main variables. It shows that the skewness and kurtosis values for all variables ranged between −1 and +1, indicating the normal distribution of the data and allowing the use of Pearson's correlation test.

**TABLE 1:** Description of main variables and assessment of convergent validity.

Variable	M	SD	Skewness	Kurtosis	AVE	CR
CRF	23.26	15.20	0.66	−0.34	0.94	0.97
FCR	62.54	26.38	0.39	0.26	0.51	0.88
Psychological distress	14.43	11.51	0.60	−0.81	0.70	0.96
Rumination	22.45	14.37	0.50	−0.52	0.82	0.93
Insomnia	12.28	6.80	0.26	−0.52	0.68	0.94

Abbreviations: AVE, average variance extracted; CR, composite reliability; CRF, cancer‐related fatigue; FCR, fear of cancer recurrence; M, mean; SD, standard deviation.

Significant positive correlations were observed among the key study variables (*p* < 0.05). CRF and psychological distress exhibited the strongest correlation with insomnia (*r* = 0.55). Additionally, all three independent variables (CRF, FCR, and psychological distress) showed significant positive relationships with the mediating variable of rumination, indicating substantial associative links which are detailed in Table 2.

**TABLE 2:** Correlation matrix among research variables and test for divergent validity.

Variable	1	2	3	4	5	VIF
1. CRF	0.97					2.75
2. FCR	0.52*	0.71				2.12
3. Psychological distress	0.74*	0.54*	0.83			3.62
4. Rumination	0.75*	0.59*	0.72*	0.90		3.54
5. Insomnia	0.55*	0.37*	0.55*	0.49*	0.82	

*Note:* The underlined numbers indicate the results of discriminant validity using the Fornell‐Larcker method.

Abbreviations: CRF, Cancer‐related fatigue; FCR, fear of cancer recurrence; VIF, variance inflation factor.

* *p* < 0.05.

The SEM technique using the PLS approach was employed to test the conceptual model. The model confirmed six out of seven hypothesized paths, demonstrating the significant mediating role of rumination. However, the direct path from FCR to insomnia did not show statistical significance (*p* > 0.05), emphasizing the importance of indirect effects through rumination, all of which were statistically significant (*p* < 0.05). The model fit was assessed using various indices, including the chi‐square test of model fit (*χ* ^2^ = 500, DF = 418, *p* < 0.05). The model's fit was robust, with a coefficient of determination for insomnia at 0.621, indicating that 62.1% of the variance in insomnia was accounted for by the model variables. Figure 1 illustrates the standardized coefficients of the tested model.

Moreover, the Q2 (CV‐Redundancy) index for insomnia was higher than 0.15, at 0.357, suggesting a suitable value and indicating that the structural model has a high and appropriate fit according to the redundancy index. The Goodness‐of‐Fit value, measuring the model's overall fit, was 0.61, higher than the benchmark of 0.36, suggesting the model had a suitable fit. Overall, the fit indices of the empirical model confirmed the conceptual model. Table 3 presents the test results of the model relationships, where direct and indirect effects were calculated using coefficients from SEM, and the significance of mediating relationships was assessed using the bootstrapping method. The results indicate that, with at least a 95% confidence level, the direct effects of CRF, psychological distress, and rumination on insomnia were confirmed, and the direction of all these relationships was positive (*p* < 0.05). The direct effect of FCR on insomnia was not confirmed (*p* > 0.05). The assessment of indirect effects showed that all mediating relationships were validated, and rumination significantly mediated the relationship between CRF, FCR, psychological distress, and insomnia (*p* < 0.05). The analysis of effect strength revealed that rumination had the strongest direct effect on insomnia with a coefficient of 0.427, psychological distress had the strongest indirect effect with a coefficient of 0.218, and the strongest overall impact was from psychological distress with a coefficient of 0.557. Overall, the model testing results indicate that FCR affected insomnia only indirectly, while CRF and psychological distress impacted insomnia both directly and indirectly through the mediation of rumination.

**TABLE 3:** Results of structural model relationship and assessment of direct, indirect, and total effects on insomnia.

Path	Direct effect		Indirect effect		Total effect	95% CI (direct)	95% CI (indirect)
	Standard coefficient	*p*‐value	Standard coefficient	*p*‐value			
CRF → insomnia	0.356	<0.001	0.106	0.002	0.462	0.290–0.422	0.038–0.174
FCR → insomnia	0.080	0.421	0.073	0.016	0.153	−0.034–0.194	0.026–0.120
Psychological distress → insomnia	0.339	<0.001	0.218	<0.001	0.557	0.265–0.413	0.146–0.290
Rumination → insomnia	0.427	<0.001			0.427	0.351–0.503	−

Abbreviations: CI, confidence interval; CRF, cancer‐related fatigue; FCR, fear of cancer recurrence.

## 4 DISCUSSION

Our study aimed to model the structural equations of insomnia in cancer survivors based on CRF, FCR, and psychological distress, with a focus on the mediating role of rumination. The results demonstrated that CRF and psychological distress have significant direct and indirect effects on insomnia, mediated by rumination. Furthermore, FCR did not have a direct effect on insomnia but influenced it indirectly through rumination. These findings align with the results reported by Haque et al.^5^ Otte et al.^29^ Hwang et al.^30^ Thorsteinsson et al.^31^ and Slavish et al.^32^

According to the model by Harvey et al.^49^ individuals with sleep disorders suffer from repetitive thinking. These recurrent thoughts primarily focus on concerns about insufficient sleep, not functioning well during the day, experiencing daytime symptoms of insomnia, experiencing fatigue, lethargy, disturbed mood, and cognitive problems such as difficulties in attention and concentration. This category of negative repetitive thoughts can lead to the emotional arousal of the individual, perpetuating insomnia.^50^ According to this model, repetitive thinking observed during the night and before sleep can also occur during the day. It can activate emotions associated with insomnia, such as feelings of turmoil caused by insomnia, thoughts about daily fatigue, and considerations about the cycle of sleep and tiredness, exacerbating the problem at night. This situation intensifies negative mood and psychological distress during the day, which in turn further activates the sympathetic nervous system, leading to chronic hyperarousal, compounded fatigue, and FCR as a recurring thought content, culminating in insomnia. In line with this perspective, Guastella and Molds^51^ believe that difficulties in regulating emotions and intrusive thoughts, leading to heightened night‐time arousal and the adoption of negative emotion‐regulation strategies like catastrophizing, directly affect sleep quality.

In a qualitative study examining the sleep patterns of individuals with breast cancer, Hwang et al.^30^ concluded that sleep problems begin with the onset of the disease and its treatment process, remaining persistent for up to 5 years post‐illness. The individuals attributed this problematic sleep cycle to factors such as anxiety, rumination, and everyday stress. Thorsteinsson et al.^31^ showed that the combination of stress, anxiety, and depression, which together represent psychological distress, culminates in rumination. This element subsequently leads to a disruption in the night‐time sleep rhythm and a decrease in mental sleep quality, resulting in impaired daily functioning, delayed sleep onset, and eventually sleep disorders, exacerbating daytime fatigue. Thus, rumination markedly exacerbates stress and psychological distress, ultimately leading to poor sleep quality and increased fatigue. Furthermore, Slavish et al. in their model, showed that rumination acts as a mediator between depressed mood, sleep quality, and mental health.^32^ It functions as a psychological mechanism through which negative mood leads to sleep disorders.

Although the results of the mentioned studies align with our research findings, our study sample consisted of cancer survivors, marking a distinct difference from the majority of the samples in previous research. In interpreting the results, it's apparent that cancer treatment, despite increasing patient survival with interventions like radiation and chemotherapy, also causes various long‐term consequences.^4^ These include cognitive problems, fatigue,^16^ worries about the side effects of the disease, and FCR.^18^ These factors can be distressing in multiple ways for individuals, leading to psychological distress,^19^ which in turn exacerbates FCR, anxiety, and rumination. Consequently, cancer survivors harbor thoughts with negative emotional content about various aspects of their illness and life. They continuously and passively tend to focus on their distressing symptoms, their underlying causes, and the potential implications of these symptoms. These experiences, by initiating rumination, lead to the development of insomnia.

According to Morin et al.^7^ when individuals have a high biological predisposition for arousal, they experience increased irritability and physiological arousal in response to anxiety‐inducing situations and circumstances. Insomnia manifests as one of the consequences of this heightened arousal. Furthermore, studies indicate that a biological predisposition for arousal and responsiveness to stress affects how individuals cope with psychological stressors. This intensifies the level of cognitive arousal stemming from such situations, leading to heightened physiological arousal and, eventually, insomnia. Anxiety, as another significant component of psychological distress, chronically contributes to insomnia. Research has shown that simple anxieties such as situational anxiety, changes in the sleeping environment, and chronic anxieties like fear of nightmares, muscle tension, and the formation of intrusive thoughts centered around nighttime sleep can act as triggers for the development of insomnia in individuals. As a result, these individuals face difficulties in initiating and maintaining sleep, experiencing frequent awakenings, and subsequently suffer from fatigue and sleepiness throughout the day.^52^

Furthermore, the relationship between insomnia and psychological distress is bidirectional. Psychological distress can cause insomnia, and insomnia can similarly amplify psychological distress. In line with this, Glozier et al. demonstrated that shorter sleep duration linearly exacerbates psychological distress. Each hour of reduced sleep increases the risk of experiencing psychological distress. Conversely, a short sleep duration is a risk factor for the persistence of psychological distress in individuals over the following year.^53^

Given the significant role of rumination in perpetuating insomnia among cancer survivors, it is crucial to focus on interventions that specifically target ruminative thoughts. Cognitive Behavioral Therapy for Insomnia (CBT‐I) is the gold‐standard treatment for insomnia and has been shown to be effective in reducing rumination and improving insomnia.^54^ It follows a comprehensive protocol that includes five crucial skills: cognitive therapy, sleep restriction, sleep hygiene, stimulus control, and relaxation training.^55^

Furthermore, augmenting CBT‐I with components specifically designed to address rumination may enhance its effectiveness.^36^ For instance, incorporating mindfulness‐based cognitive therapy can help individuals develop awareness of their thoughts and reduce the impact of rumination on their insomnia.^56^ Mindfulness techniques teach patients to observe their thoughts without judgment, thereby reducing FCR and the automatic and negative response to ruminative thoughts.^56^, ^57^

In addition to CBT‐I, metacognitive therapy (MCT) can be particularly beneficial for cancer survivors experiencing high levels of rumination.^58^ MCT focuses on changing the beliefs and processes that sustain repetitive negative thinking, such as rumination.^59^ By addressing the underlying cognitive processes, MCT can help reduce rumination and improve insomnia. Integrating these cognitive interventions into routine cancer care can provide holistic support for cancer survivors. This can involve training healthcare providers in delivering CBT‐I and MCT, as well as developing digital health interventions that make these therapies more accessible.

This study had several limitations that must be acknowledged in the interpretation of its results. First, our study's cross‐sectional design limits our ability to infer causal or predictive relationships between the variables under consideration. Our findings reveal associations that could guide future longitudinal research, which is required to establish causality and investigate the dynamics of these relationships over time. Consequently, causal conclusions regarding the predictive factors cannot be drawn from our study. Therefore, it is recommended that future studies investigate the role of psychological factors in insomnia using more controllable methods such as experimental and comparative causal research.

Second, the proportion of female to male participants in our study (88.5% female and 11.5% male) is notable and reflects the gender distribution commonly observed in cancer survivorship research. This disproportionate representation could potentially impact the generalizability of our findings. Women are more likely to report higher levels of psychological distress and sleep disturbances compared to men, which might have influenced the observed relationships among CRF, FCR, psychological distress, rumination, and insomnia. Future studies should aim to include a more balanced gender distribution to explore potential gender differences in these relationships more comprehensively. Additionally, the inclusion of gender as a moderating variable in future analyses could provide deeper insights into how gender impacts the pathways between psychological factors and insomnia in cancer survivors.

Furthermore, since this study was conducted on a population of cancer survivors, caution is needed in generalizing its findings to other populations. Future experimental research should focus on evaluating the effectiveness of cognitive and metacognitive interventions for reducing rumination and psychological distress and assess their effects on the insomnia of cancer survivors. These studies should also compare and analyze the factors examined in this study with those in populations currently undergoing treatment, non‐affected individuals, and other individuals with physical diseases to explore and compare the prevalence of these issues among these groups.

Finally, it is also important to recognize that other medical factors, such as cancer type, stage, and prognosis, could affect patients' experiences of the observed symptoms. Although our study controlled for some of these variables, future research should consider these factors in more detail to provide a more comprehensive understanding of their influence on insomnia in cancer survivors.

## 5 CONCLUSION

The findings of our study indicate that CRF, FCR, and psychological distress significantly impact insomnia in cancer survivors. Our research highlights the pivotal role of rumination as a mediating factor in the relationship between these variables and insomnia. Our study reveals a novel finding regarding the significant role of rumination in the relationship between CRF, FCR, and psychological distress in perpetuating insomnia in cancer survivors. This finding not only aids in better understanding the mechanisms of this process but also highlights the crucial role of this factor in the formation and continuation of insomnia. It underscores that psychological interventions for treating this issue should pay special attention to the significant role of rumination and employ appropriate techniques to reduce rumination. Considering the negative consequences of insomnia among cancer survivors, the development and implementation of new and accessible therapeutic protocols, as well as conducting simple and affordable educational and therapeutic interventions aimed at addressing psychological distress, FCR, CRF, and particularly rumination, could pave the way for improving insomnia in cancer survivors and reducing the adverse outcomes associated with this issue.

## AUTHOR CONTRIBUTIONS

**Omid Amani:** Conceptualization (equal); data curation (equal); formal analysis (equal); methodology (equal); writing – original draft (equal). **Mohammad Ali Mazaheri:** Conceptualization (equal); data curation (equal); formal analysis (equal); methodology (equal). **Mona Malekzadeh Moghani:** Resources (equal). **Fariba Zarani:** Resources (equal). **Rasool Hamidi Choolabi:** Conceptualization (equal); methodology (equal); resources (equal); writing – original draft (equal).

## FUNDING INFORMATION

Funding sources played no role in the study design, data collection, data analysis, data interpretation, or report writing.

## CONFLICT OF INTEREST STATEMENT

The authors declared that there are no conflicts of interest.

## ETHICS STATEMENT

The Medical Ethics Committee of Shahid Beheshti University of Medical Sciences, Tehran, Iran (Ethical approval code: IR.SBMU.CRC.REC.1402.001) approved the study protocol.

## PATIENT CONSENT STATEMENT

Informed consent was obtained from all participants included in this study. The participants were fully informed about the nature, purpose, and potential risks of the research, and their rights to withdraw at any stage without consequence were emphasized. All data were anonymized to protect participant privacy and confidentiality.

## CONSENT FOR PUBLICATION

Publishing was agreed upon by all authors.

## Data Availability

The data that support the findings of this study are available from the corresponding author upon reasonable request.
